# Using Pathway Signatures as Means of Identifying Similarities among Microarray Experiments

**DOI:** 10.1371/journal.pone.0004128

**Published:** 2009-01-06

**Authors:** Luca Beltrame, Lisa Rizzetto, Raffaele Paola, Philippe Rocca-Serra, Luca Gambineri, Cristina Battaglia, Duccio Cavalieri

**Affiliations:** 1 Department of Pharmacology, University of Firenze, Firenze, Italy; 2 Institute for Biomedical Technologies, National Research Council, Milano, Italy; 3 European Bioinformatics Institute, Cambridge, United Kingdom; 4 Inspect.it, Arezzo, Italy; 5 Department of Science and Biomedical Technologies, University of Milano, Milano, Italy; Minnesota State University Mankato, United States of America

## Abstract

Widespread use of microarrays has generated large amounts of data, the interrogation of the public microarray repositories, identifying similarities between microarray experiments is now one of the major challenges. Approaches using defined group of genes, such as pathways and cellular networks (pathway analysis), have been proposed to improve the interpretation of microarray experiments. We propose a novel method to compare microarray experiments at the pathway level, this method consists of two steps: first, generate pathway signatures, a set of descriptors recapitulating the biologically meaningful pathways related to some clinical/biological variable of interest, second, use these signatures to interrogate microarray databases. We demonstrate that our approach provides more reliable results than with gene-based approaches. While gene-based approaches tend to suffer from bias generated by the analytical procedures employed, our pathway based method successfully groups together similar samples, independently of the experimental design. The results presented are potentially of great interest to improve the ability to query and compare experiments in public repositories of microarray data. As a matter of fact, this method can be used to retrieve data from public microarray databases and perform comparisons at the pathway level.

## Introduction

Since their first inception a decade ago, microarray studies have become widely used in the research community, thanks to their ability to assess the expression of thousands of genes in a single laboratory event. The belief that such wealth of genomic information the community could not afford to lose has led to the development of microarray standards [1], [2] and databases including two major public microarray repositories, Gene Expression Omnibus (GEO) [3] and ArrayExpress [4], in the hope of enabling mining and exploration of newly acquired data space.

Identifying biologically meaningful information in relatively noisy data represents a significant tasks and so far breakthrough have been few and far between. Comparisons made on the level of gene lists obtained by different statistical methods or from different datasets hardly converge [5]. As a consequence, the usefulness of the vast amounts of data stored in public repositories is subject to debate. At the same time, it is becoming important to use more than a single data set when analyzing microarray data [6] and gather hundreds or thousands of samples to develop prognostic markers [7]. Reaching such a goal is difficult when information obtained from different experiments do not overlap. This is mainly because the data often has been generated with different microarray platforms, hybridization protocols, and the authors use different methods and different thresholds to calculate differentially expressed genes (DEGs) [8] . Finding ways to reliably compare different microarray data sets is therefore important to obtain biologically sound and reproducible information from different datasets.

In the past years collections of all the differentially expressed genes in a given condition that exclusively characterize that condition, have been proposed as “gene signatures” for a condition [9]–[11]. However, the reliability and reproducibility of such signatures has been questioned [12], [13], because of the influence of the statistical assumptions used, or errors in the methodology.

The number of inconsistencies and discrepancies when microarray data sets are compared are often reduced when approaches that take into account biologically related sets of genes, rather than single entities, are used [14]. The first methods developed with this approach aimed at identifying significantly under- or over-represented terms in the Gene Ontology [15], [16]. A second approach instead focuses in identifying significantly expressed gene sets (sometimes incorrectly referred as pathways or cellular networks) in a given condition using different statistical measurements: Z-score [17], Gene Set Enrichment Analysis [18], signed Fisher Exact Test [19], Global test [20] and impact analysis [21].

Recently we developed a bioinformatic environment called Eu.Gene [22] containing a repository of all the freely available biological pathways and different statistical methods dedicated to analyze expression datasets and assess for enrichment in biological pathways. EuGene relies on 2 components, (i) a database of consistently annotated pathways collected from a number of state of the art pathway resources and (ii) a module of computation implementing an array of tailored heuristics and statistical methods.

We developed a method allowing to assess similarity of samples in microarray databases. To this aim we used EuGene to generate “Pathway Signatures”, recapitulating the biologically meaningful pathways related to some clinical/biological variable of interest, and used them in an analysis workflow to compare different microarray experiments.

## Results

### Selection of the data sets

We decided to use as reference experiments two distinct data sets: firstly, a data set of gene expression experiments on a model organism, *Saccharomyces cerevisiae* (*S. cerevisiae*) using the dataset from the Rosetta compendium of deletion mutants [23], and secondly 106 experiments (grouped in 7 sets) focused on the exposure of *Homo sapiens* dendritic cells (DCs) to different stimuli.

Following up the data set collection, data were pre-processed and subject to pathway analysis (see Methods). Then, we formulated two predictions basing on biological evidence against which the method would be tested. With regards to the yeast deletion mutants, we predicted that the *ssn6* and *tup1* mutants would be similar, as acting as part of the same protein complex. Regarding the dendritic cells data set, we performed a wet lab experiments stimulating DCs with cells of the yeast *S. cerevisiae* in exponential growth phase (analyzed in the same manner as the public data) and we predicted that this experiment would show similarity with the samples from GEO data set GSE6965 (gene expression profile of monocyte-derived DCs stimulated for 6 h with germinating germ tubes of *Aspergillus fumigatus*).

### Statistical methods

We initially compared different statistical approaches for pathway signature generation. To address the issue of the most appropriate statistic, we evaluated both Fisher's Exact Test (FET) and Gene Set Enrichment Analysis (GSEA), two well-established methods for pathway analysis. As GSEA required samples to be divided into two distinct classes, it was not performed on the yeast data. On the other hand both GSEA and FET were used with the DC samples. FET produced results in the form of signed p-values, while GSEA produced signed enrichment scores (ES).

Yeast data was in the form of log_10_ ratios and thus, after a conversion to log_2_, was used directly for the Fisher's Exact Test.

In order to use dendritic cell data for the Fisher's Exact Test, we converted the Affymetrix absolute expression values into ratios (see Methods). When dealing with Affymetrix data, we had the choice of cross-normalizing all the data sets, or to perform normalization independently for each of them. Initially we compared the two approaches. We calculated the ratios from either the comparison between the treated samples against a common baseline (median expression of all control experiments) or by doing paired ratios between each treated sample and its corresponding control. Between those two approaches, the paired ratios performed better (data not shown) and was therefore used in all subsequent analyses both to prevent a “smoothing” effect of the differences between the data sets themselves, and to make sure that we could add more samples without having to normalize the data sets again.

In order to selecting the most representative FET threshold, FET thresholds were selected from the 2σ interval of the binomial distribution of the expression values.

When using FET, we addressed the question of how to use the collection of p-values for a defined set of pathways to generate a pathway signature with the aim of comparing different microarray experiments. Firstly, we corrected the p-values for multiple testing (see Methods) then our first attempt was to transform the p-values into a measure whose magnitude would express the degree of significance, and to that purpose, we transformed p-values into Pathway Enrichment Factors (PEFs). Secondly, we transformed p-values in signed binary enrichment factors (sBEFs) (see Methods): these factors categorized p-values into three classes (significantly up-regulated, significantly down-regulated, and not significant).

On the other hand, GSEA enrichment scores (ES) were used directly, without any modification.

We then evaluated the performance of these metrics (sBEF or enrichment score), as expressed in the ability of assessing similarity according to the expectation that biologically similar experiments should show identical or similar pathway profiles (defined as collections of PEFs, sBEFs or enrichment scores for the pathways used for testing).

### Selection of methods to compare sets of p-values and/or enrichment scores

To find reliable ways to group samples together, we investigated a number of unsupervised hierarchical clustering techniques that would be used to group together the metrics we had selected. For this purpose, Unweighted Pair Group Method with Arithmetic mean (UPGMA) clustering, standard hierarchical clustering, and hierarchical clustering with support trees were used (Figures S1, S2, S3, S4, S5). In the last case we bootstrapped samples and pathways over 100 and 1000 iterations. Pathways and samples were clustered with different metrics: in particular, Euclidean distance, Pearson's correlation and Manhattan distance were used. We ranked the clustering methods based on the ability to group biologically similar samples together. We found hierarchical clustering with support trees to be the most reliable method, and that 100 iterations were sufficient to obtain reproducible results. Although all the distance metrics performed equally in grouping biologically similar samples, we found Euclidean distance to give the best visualization of the data.

Additionally, as sBEFs were categorical values, we also evaluated the similarity between samples by using a metric suited for this type of data (the Jaccard index).

### Validation of the method

**Figure 1:**
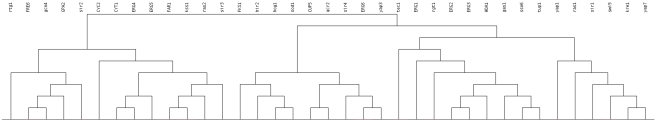
Clustering of yeast samples with PEFs using Pearson's correlation. Sample tree originated from the clustering of FET Pathway Enrichment Factors (PEFs) values with Pearson's correlation.

**Figure 2:**
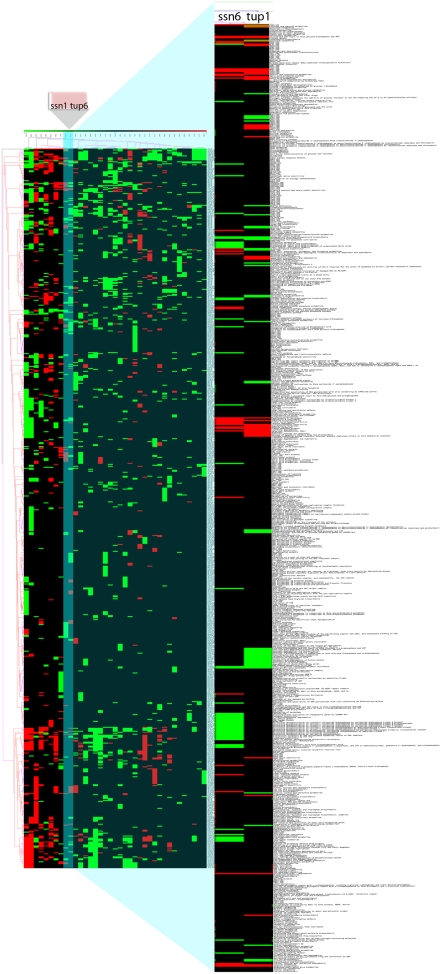
Clustering of signed Binary Enrichment Factors (sBEFs) obtained from the FET analysis on yeast samples. Colored spots indicate significant (p≤0.05) up- (red) or down- (green) regulation. The colors of the dendrogram indicate the percentages of the tree support (significance), from 50% (pink) to 100% (black). The inset shows the clustering of yeast mutants *ssn6* and *tup1*. The full figure is available as Figure S1.

With regards to yeast data, the clustering of PEFs gave results that were agreeing with our prediction, as the *ssn6* and *tup1* deletion mutants clustered together when using Pearson's correlation as clustering metric (Figure 1). The same results were observed when using sBEFs, with the difference that they were independent from the clustering metric used (Figure 2; Figures S1, S2, S3). Pathways which shared a common profile among the two samples included up-regulated alternative glucose metabolism related pathways (activation of penthose-phosphate pathway, galactose metabolism and degradation, lactose metabolism) and down-regulated glucose metabolism pathways such as transcriptional activation of glucose metabolism genes. Standard hierarchical clustering and UPGMA clustering results were also in agreement with the prediction (Figures S4 and S5). On the other hand, the Jaccard index between the two samples was 0.14, a possible indication that such a method is less powerful than clustering to detect biologically relevant similarity.

**Figure 3:**
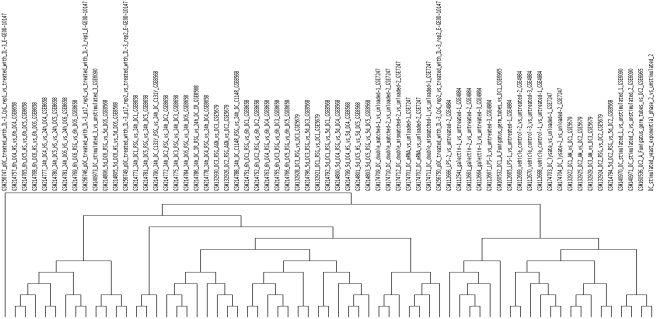
Sample clustering of Fisher's Exact Test samples on dendritic cell data. Sample tree originated from the clustering of FET signed Binary Enrichment Factors (sBEFs) values with Euclidean distance.

**Figure 4:**
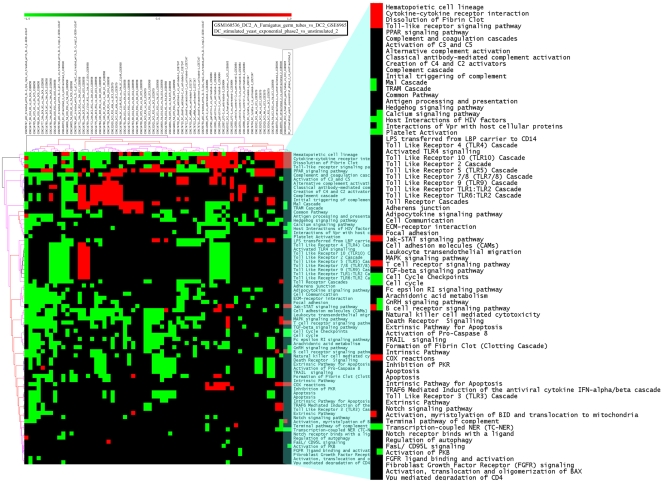
Clustering of signed Binary Enrichment Factors (sBEFs) obtained from the FET analysis on dendritic cell samples. Colored spots indicate significant (p≤0.05) up- (red) or down- (green) regulation. The colors of the dendrogram indicate the percentages of the tree support (significance), from 50% (pink) to 100% (black). The inset shows the clustering of sample GSM160356 from data set GSE6965 with the in-house dendritic cell experiment.

The results obtained with the dendritic cells data also confirmed the predictions. wet lab experiments (analyzed in the same manner as the public data) and we predicted that this experiment would show similarity with the samples from GEO data set GSE6965. The Pathway Signatures of our ad-hoc sample produced stimulating DCs with cells of the yeast *S. cerevisiae* in exponential growth phase, clustered together with gene expression profile of monocyte-derived DCs stimulated for 6 h with germinating germ tubes of *A. fumigatus* (Figure 3; Figures S6, S7). The result was even more striking than the yeast data, both sBEFs and PEFs produced results that were independent from the clustering metric used. When using PEFs, the two samples belonged to the same sub-cluster (data not shown), while with sBEFs they belonged to the same sub-cluster and were the most closely associated. Pathways which shared a similar profile among the two samples included the Toll-like receptor signaling pathway, JAK-STAT signaling, and cytokine-cytokine receptor interaction (Figure 4). Since we expect the two stimuli to elicit signaling from the same pathogen associated molecular patterns (PAMPs) these results show that pathway-level signatures identify samples with similar biological characteristics. Standard hierarchical clustering and UPGMA clustering results were also in agreement with our prediction (Figures S8 and S9). The Jaccard index between our experiment and sample GSM160356 showed a good similarity (0.75) and the other replicate from data set GSE6965 showed even greater similarity (0.9). These findings show that FET pathway based metrics are a powerful tool to identify similarity between experiments.

**Figure 5:**
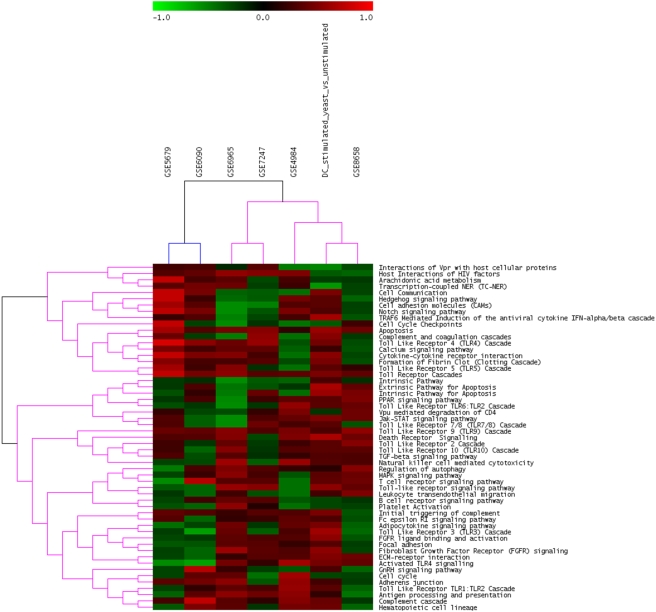
Clustering of Enchriment scores obtained from GSEA run on the dendritic cells data sets. Red indicates up-regulation, while green signifies down-regulation. The colors of the dendrogram indicate the percentages of the tree support (significance), from 50% (pink) to 100% (black).

On the other hand, when we clustered enrichment scores from GSEA on the dendritic cell data, results were not in agreement with the prediction (Figure 5): our experiment clustered with the GSE8658 data set (response to different ligands), while GSE6965 clustered with GSE7247 (an experiment on loading DCs with different antigens). These findings indicate that FET transformed p-values are more effective in finding similarities among different experiments when compared to GSEA enrichment scores.

## Discussion

In this work we propose a novel method to compare microarray experiments at the pathway level, our work addressed three major tasks: (i) defined a metric measuring the probability of a set of pathways to be related to some clinical/biological variable of interest and the relative importance of that set in the context of the biological problem, (Pathway Signature) (ii) proposed a method using these pathway signatures to assess relative similarity of the experiments ; (iii) proved the validity of this method applying it to two different well defined biological problems on two independent collections of microarray experiments.

As a proof of concept we selected datasets representing well known stimuli in well known biological systems. One of the datasets was from *S. cerevisiae*, the most extensively studied model organism, with a well characterized genome where all the genes are represented on the array, widely used to test bioinformatics methods. We selected the Rosetta compendium of deletion mutants [23] as it measures the steady state response to a very precise alteration, the deletion of a given gene, and consequently is relatively simple to associate a phenotype to a particular pathway profile. As second dataset we decided to use data sets from *Homo sapiens* because the majority of the experiments stored in GEO and ArrayExpress are from human samples. We chose transcriptomic datasets from DCs as they have some clear advantages respect to other fields. Firstly, the cell type is well-defined, which enables the study of the alterations in gene expression following stimulation. Secondly, there is the possibility to perform prediction and hypothesis-driven functional genomics studies aiming at the reconstruction of the networks of molecular interactions characterizing specific DC differentiation programs.

The aim of our work was to propose a procedure to assess similarity between microarray experiments at the pathway level generating “Pathway signatures” (PS) for a set of experiments and use these signatures to interrogate microarray databases.

The need for better methods to identify similarities in microarray data sets arises from the fact that although the analysis techniques have constantly improved over the past years, one of the biggest hurdles remains the comparability among distinct data sets produced by different researchers and laboratories, resulting in lists of genes which do not overlap, or overlap in a very limited fashion. A possible reason relies on the different assumptions on the data used by different statistical methods [8]. This is a strong limitation, because identifying biologically similar samples would increase the power and the reproducibility of a study, especially since in most studies the number of samples can be a limiting factor, which could be compensated using already published experiments.

Also, as the number of publicly available data sets increases (at the time of writing, GEO and Array Express host a total of 221,815 and 110,356 hybridizations, respectively), it is important to have reliable method to compare microarray data from different sources.

The improvements observed when comparing different experiments at the pathway level [14] is coherent with the assumption that genes never act alone in a biological system, but participate in a cascade of networks, an approach overlooked by gene-based analyses.

The selection of the statistical method used to measure enrichment is central to our approach.

Different metrics have been proposed to integrate the probability of alteration of a sector of the cellular network (pathway) and the relative importance of that pathway in the context of the biological problem, such as the probability vector [19], the impact factor (IF) [21]. Other methods have been devised for the identification of regulatory modules and their regulation program by integrating genome-wide location and expression data [24]–[27]. However, to our knowledge, these methods have not been employed to compare a large number of experiments assembled from different microarray data sets.

FET p-values had to be transformed to improve interpretation: p-values express a probability, and the smaller they are, the more significant the result is, while from a conceptual point of view it is better to express pathway enrichment as a number that it is either categorical or the more significant the greater it is. That is the reason we first used the logarithm of reciprocal of the p-value (the Pathway Enrichment Factor), to express the measure in a scale that would avoid interpretation problems, the PEF was finally obtained multiplying the value for a sign representing the “direction” of a pathway, with the same approach described in [19]. Our results showed that clustering PEFs grouped samples according to their biological similarity. In yeast we observed as part of the same cluster transcriptional profiles of the deletion mutants of, *ssn6* and *tup*, genes that form a co repressor complex which is responsible for repressing a large number of *S. cerevisiae* genes, including glucose-responsive genes, DNA damage genes and oxygen utilization genes [28], [29]. As *ssn6* and *tup1* act together, deletion of one or the other gene is expected to yield a similar behavior. On the yeast dataset the exact topology of the cluster was dependent on the metric used. This is quite understandable: sets of PEFs can be noisy, as there are all the possible ranges of p-values, and identifying which pathways were “significant” and which “not significant” was not always straightforward. This can influence the clustering, and so certain metrics prove to be more useful than others, for example Pearson's correlation was the metric which most correctly grouped *ssn6* and *tup1* in our yeast data. This result could be biologically relevant, as although ssn6 and tup1 are part of a complex, tup1 has also a function alone, and as a matter of fact, the pattern of significantly altered pathway (shown by their sBEFs) in the two deletion mutants exhibits differences. This biological difference could result in changes in the direction in which some pathways are affected by one deletion or by the other. Alternatively this result could reflect greater sensitivity to technical “noise” in the data of some of the metrics used.

Biclustering of PEFs on the dendritic cell data sets gave concordant and biologically relevant results, the responses elicited by stimulation with *S. cerevisiae* follow the same downstream signalling as the ones in response to the fungus *A. fumigatus* [30]–[33] their PEFs clustered one next to the other with all the metrics used.

On the other hand, sBEFs produced consistent results from a clustering point of view, both for the yeast and the DCs datasets, independently from the clustering method, thus making very easy the identification of similar datasets.

We selected sBEFs to produce the PS as they can be easily used as a “barcode” that can uniquely attached that sample, facilitating “querying” a database of PSs.

The observation that sBEFs, PEFs and PSs, identify similarity between samples that are biologically meaningful, indicates that the categorical transformation of the p-values does not affect the observed result, concluding that the observed similarity has a biological meaning rather than resulting from a manipulation of the data.

When testing different methods to compare experiments at the pathway level, we initially chose clustering because of its flexibility and robustness in representing biological data. The type of clustering used was biclustering with support trees over 100 or 1000 iterations [34] as implemented in TMeV [35], because the clustering results obtained with this method are more statistically sound as they do not depend on the original order of the genes and the pathways. Also, clustering has been applied successfully to gene expression studies [36]–[39]. From an ontological point of view the sBEFs can be considered as categorical phenotypes, thus clustering is a legitimate approach to classify “omics” data, as it has been used on datasets of both continuous and categorical data, such as human haplotypes in population studies [40], [41] and phenotypic observations, such as lord Fisher's *Iris* data set [42], probably one of the most widely used data sets used in clustering and pattern recognition studies.

Yet since sBEFs are categorical rather than continuous, we investigated the use of an alternative metric (the Jaccard index), since biclustering may not be completely suited for this type of data. Our results showed an excellent agreement with the clustering for DC data, but some inconsistencies with the yeast data, in agreement with the clustering of the PEFs for this dataset. This could reflect some biological instances, or alternatively could be related to noise deriving from technological issues, as the yeast dataset is a none year old two color array. Another possibility is that measures like the Jaccard index may not be the most robust to capture the inherent complexity of “omics” data. In any case, our future work will be aimed at improving the grouping metrics for sBEFs.

Results with GSEA on the dendritic cell data did not satisfy our prediction, due to the different numbers of experiments present in the different classes and the difficulty to classify one data set in a class or another. In fact, the grouping observed with FET scores on DC data was absent when using GSEA data. There are two possible reasons to this inconsistency. First of all, GSEA compares two groups (treated versus untreated) as opposed to single-sample analysis performed with FET. Thus, the effect of inter-donor variability (all data sets have at least two biological replicates for each sample) is noticeable and is not corrected by the pathway approach. The second reason is the heterogeneity of the samples themselves, that are subject to different treatments. As a result, GSEA rounds everything to the lowest common denominator, presenting an “average” profile where individual differences are smoothed out. Also, the number of control and treated samples in each data set is different (this is usually the norm when comparing different data sets), so there is an imbalance among the various GSEA analyses.

We can conclude that the ability to investigate at the single sample makes the Fisher's Exact Test conceptually more appropriate to search for similarities among experiments in a microarray databases, that contain a number of hybridizations that should be interrogated without necessarily specifying the membership to a data set or another. Lastly, GSEA requires dividing the samples in two distinct phenotypic classes in order to operate, and division in classes is not necessarily straightforward. As a matter of fact, such a type of analysis is not suitable for two-color microarrays (which present data as a ratio between treatment and control), and therefore we were not able to use it with our yeast data set.

Overall our results show that using pathway signatures in conjunction with hierarchical clustering with support trees is a powerful and useful technique to compare experiments produced by different people and laboratories with greater power than with the traditional analysis techniques. The results are also easier to interpret and discover biologically meaningful implications, making this approach an ideal candidate to analyze data from different sources. PS generated using sBEFs can be useful as “barcodes” to classify experiments in microarray databases and clustering of sBEFs can be a useful way to query experiments in databases according to their similarity at a pathway level. Thus, we propose to store PSs as an additional experimental annotation in the microarray databases and implement methods using pathway signatures to query experiments in public databases and concurrent analyses of subsets of experiments.

Despite the effectiveness of the method, there are still some drawbacks that will need to be addressed in the future. First of all, Fisher's Exact Test result depend on the lower and upper cut-offs for expression, and at the current time they are defined by the user. We were well aware of the limitations of this method but also aware of its robustness if the appropriate threshold is used, in agreement with the findings from Bussemaker and Boorsma, that proved that the Fisher's Exact Test outperforms other metrics when appropriately selecting the threshold [43]. Thus, we calculated the appropriate cut-offs automatically, basing on the 2σ interval of the binomial distribution of the expression values [44], and we will implement this method directly in a future version of Eu.Gene. Secondly, both FET and GSEA make the assumption that genes in a pathway are independent from each other, which is clearly not the case in real biological system. An alternative system with the potential to dramatically improve the results of pathway analysis has been recently proposed [21], which keeps track of the interdependence of genes. However, its use is currently limited to signal transduction pathways from KEGG and requires an accurate description of causality among the events, which is not the case for the vast majority of the pathways present in public resources. We have partially incorporated this idea of direction by calculating signs attached to the p-values. We plan to increase the sophistication of the method, implementing proper directionality of the events using curated pathway sets with a consensus on the number, type and order of the connections between the members of the pathway. With the ability to include directionality, the application of methods for module network analysis using improved PSs instead of genes [45] holds the promise to unravel the hierarchical structure in the control of the cellular pathways and reconstruct the modular structure determining changes in a transcriptional profile.

## Methods

### Data sets

#### Yeast data sets

A selection of 37 experiments on yeast deletion mutants were obtained from the Rosetta compendium [23] . Prior to analysis, data (in the form of log_10_ ratios) were transformed into log_2_ ratios.

#### Dendritic cell data sets

106 microarray samples, corresponding to 6 data sets, were retrieved from the Gene Expression Omnibus (GEO) database (Table 1). During the data selection process, only samples hybridized on Affymetrix GeneChip HG-U133 Plus 2.0 were selected. Experimental and sample data were then downloaded using a script written in R (http://www.r-project.org) which made use of the Bioconductor package GEOquery (http://www.bioconductor.org). Raw data (CEL files) were then downloaded and extracted using custom Bash shell scripts on the Linux operating system.

**Table 1:** Dendritic data set summary.

GEO ID	Description	Type	Sample no.	Reference	Year
GSE8658	PPARg regulated gene expression in human dendritic cells	Ligand response	63	[49]	2007
GSE7247	Dendritic Cells Compare the Similarity of Endogenous and Exogenous Antigens	Loading methodology comparison	10	[50]	2006
GSE4984	Monocyte Derived Dendritic Cell Maturation	Dendritic cell maturation	12	[51]	2006
GSE6090	DC-SIGN initiates an immature dendritic cell phenotype triggering Rho activation that is used by HIV-1	Receptor signalling	6	[52]	2007
GSE6965	Gene expression profiling of human dendritic cells after infection with A. fumigatus	Dendritic cell activation	4	[53]	2008
GSE5679	Comparative gene expression profile of PPARg and RARa ligand treated human dendritic cells	Ligand response	11	[54]	2008

Dendritic cells data sets obtained from Gene Expression Omnibus (GEO).

#### In-house stimulated dendritic cell samples

Peripheral blood mononucleated cells (PBMCs) were isolated from buffy coats by density gradient centrifugation using Biocoll (Biochrom). CD14+ monocytes were isolated from PBMCs by positive selection using MACS anti-CD14 microbeads and a Midi-MACS® magnetic cell sorting device (Miltenyi Biotec, Bergisch- Gladbach,Germany). Cells were cultured in RPMI 1640 medium (Gibco BRL) supplemented with 2 mM L-glutamine (Sigma), 1% (v/v) non-essential amino acids, 100 mM sodium pyruvate, 50 U/ml of penicillin and 50 mg/ml of streptomycin (Gibco BRL) containing 10% (v/v) FCS (Hyclone). Differentiation of monocytes into dendritic cells was promoted by addition of granulocyte-macrophage colony-stimulating factor (GM-CSF 1000 U/ml, Chemicon)and recombinant IL-4 (1000 U/ml, R&D Systems) for 5 days. 3*10^6^ DCs were either not stimulated or stimulated with yeast grown in exponential phase. Cells were harvested after 4 hr of stimulation.

RNA extraction was done with TRIzol reagent (Invitrogen). Sample pre-processing and biotin labeling were performed using the Affymetrix GeneChip® cDNA Synthesis Kit and IVT Labeling Kit (Affymetrix) according to the manufacturer's protocols. Microarray were then hybridized on Affymetrix GeneChip® HG-U133A 2.0 microarrays, and scanned according to the manufacturer's instructions on a GeneChip® Scanner 3000 (Affymetrix).

Extraction, hybridization and scanning were performed by the Genopolis consortium (University of Milano-Bicocca, Italy). Microarray data have been submitted to GEO (accession number GSE13901).

### Pre-processing and normalization

CEL files were pre-processed and normalized with the Robust Multi-array Average (RMA) [46] procedure, in order to obtain absolute-scale expression levels for all genes, using custom chip definition files (CDFs) to update the annotation of the GeneChips, removing duplicate probes and mapping all probes to single Entrez Gene IDs, as previously described by Dai et al. [47]. Each data set was normalized separately. The computation was performed with the RMAExpress program, version 1.0 beta 10 (http://rmaexpress.bmbolstad.com), on the Linux operating system.

### Selection of the pathway set

To identify biologically meaningful changes in our data sets, we assembled two pathway sets, depending on the species. All pathways were defined as gene lists (containing genes associated with the pathway). The sets were prepared as follows:

- ***Saccharomyces cerevisiae*** **data:** All the pathways present in Eu.Gene (n = 852)
- **Dendritic cell data**: Pathways containing less than three elements were excluded. Out of the remaining pathways, we built a selection curated by experts from the DC-THERA European network of excellence, where pathways were classified by their level of immunological relevance. Out of the 1038 human pathways available , 80 satisfying the experts' criteria were selected to be part of the set.

### Pathway analysis

Eu.Gene [22] was used to perform the analysis on the selected pathway set. The analysis algorithms used were the Fisher's Exact Test (FET) [19] and Gene Set Enrichment Analysis (GSEA) [18].

### Fisher's Exact Test

Prior to analysis, the Affymetrix data was converted into log_2_ ratios using the following procedure. For each data set, treated and untreated sample data were separated, and the expression level of each gene in each treated sample was divided by the expression level of the same gene in the corresponding paired untreated sample, and the resulting ratio was log_2_ transformed, obtaining a list of log_2_ ratios for each gene in each treated sample. The transformed expression data, corresponding to 66 ratios, were then used for Fisher's Exact Test.

Yeast data, once converted to log_2_ ratios, was instead used directly, without any other modifications.

The upper and lower cut-offs for FET were determined from the 2σ interval of the binomial distribution of expression values [44].The algorithm was then run using the hypergeometric distribution without approximation. Each pathway was associated to a signed p-value and a matrix of signed p-values for all samples was obtained for each data set. In order to provide a correction for multiple testing, p-values were adjusted following the procedure by Benjamini *et al.* [48].

Resulting p-values from the FET analysis were then transformed prior to clustering (see below).

### Gene Set Enrichment Analysis

For each data set, samples were divided into treated (“Type-A”) and untreated (“Type-B”) groups and their expression measures (in absolute scale) were used directly for the analysis. GSEA was run on each data set separately, excluding pathways which contained less than 12 genes or more than 400 genes, to improve specificity and then significance of the obtained Enrichment Score was assessed by performing 1000 random permutations on the data. Enrichment scores from the analysis were then used for clustering.

### p-value transformation (FET)

The FET p-value tables from Eu.Gene were then transformed in order to be used for hierarchical clustering. Two approaches were used. Firstly, p-values were transformed into Pathway Enrichment Factors (PEF) as follows:
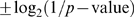
where the sign is determined by the Sign of the p-value obtained from the FET analysis in Eu.Gene.

With the second approach, p-values were converted into signed Binary Enrichment Factors (sBEFs)
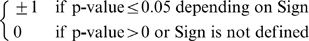
In the case of sBEFs, after transformation they were filtered to exclude non-significant pathways in all the samples (i.e., rows containing only zeroes in all samples).

### Clustering

Hierarchical clustering was performed with the TIGR Multiexperiment Viewer (TMeV), version 4.1.1 [35]. Specifically, we used standard hierarchical clustering and clustering with support trees [34]: in the latter case pathways and samples were bootstrapped over 100 and 1000 iterations. Euclidean distance, Pearson's correlation and Manhattan distance were used as distance metrics, with average linkage clustering. Both pathways and samples were clustered.

Unweighted Pair Group Method with Arithmetic mean (UPGMA) clustering was performed using Eu.Gene's internal “Path-Blast” function, creating similarity trees between different experiments in each data set with a p-value threshold of 0.05.

### Jaccard index calculation

From the sBEF matrix, the Jaccard similarity index was calculated for every possible pairing between the various sample columns. The computation was performed with the Python programming language, using the scipy-hcluster package (http://code.google.com/p/scipy-cluster/).

## Supporting Information

Figure S1 Clustering of signed Binary Enrichment Factors using Euclidean distance using support trees on yeast data. Colored spots indicate significant (p< = 0.05) up- (red) or down- (green) regulation. The colors of the dendrogram indicate the percentages of the tree support (significance), from 50% (pink) to 100% (black).
(0.94 MB PNG)

Figure S2 Clustering of signed Binary Enrichment Factors using Pearson's Correlation using support trees on yeast data. Colored spots indicate significant (p< = 0.05) up- (red) or down- (green) regulation. The colors of the dendrogram indicate the percentages of the tree support (significance), from 50% (pink) to 100% (black).
(0.92 MB PNG)

Figure S3 Clustering of signed Binary Enrichment Factors using Manhattan Distance using support trees on yeast data. Colored spots indicate significant (p< = 0.05) up- (red) or down- (green) regulation. The colors of the dendrogram indicate the percentages of the tree support (significance), from 50% (pink) to 100% (black).
(0.94 MB PNG)

Figure S4 Standard hierarchical clustering of signed Binary Enrichment Factors on yeast data. Colored spots indicate significant (p< = 0.05) up- (red) or down- (green) regulation.
(0.92 MB PNG)

Figure S5 UPGMA clustering of Fisher's Exact Test analysis results on yeast data.
(0.16 MB JPG)

Figure S6 Clustering of signed Binary Enrichment Factors using Pearson's Correlation using support trees on dendritic cell data. Colored spots indicate significant (p< = 0.05) up- (red) or down- (green) regulation. The colors of the dendrogram indicate the percentages of the tree support (significance), from 50% (pink) to 100% (black).
(0.22 MB PNG)

Figure S7 Clustering of signed Binary Enrichment Factors using Manhattan Distance using support trees on dendritic cell data. Colored spots indicate significant (p< = 0.05) up- (red) or down- (green) regulation. The colors of the dendrogram indicate the percentages of the tree support (significance), from 50% (pink) to 100% (black).
(0.22 MB PNG)

Figure S8 Standard hierarchical clustering of signed Binary Enrichment Factors on dendritic cell data. Colored spots indicate significant (p< = 0.05) up- (red) or down- (green) regulation.
(0.20 MB PNG)

Figure S9 UPGMA clustering of Fisher's Exact Test analysis results on dendritic cell data.
(0.63 MB JPG)
