# Supplementary figures and images for: Using Pathway Signatures as Means of Identifying Similarities among Microarray Experiments
Source: PLoS One. 2009 Jan 6;4(1):e4128. doi: 10.1371/journal.pone.0004128 (PMC2610483; doi:10.1371/journal.pone.0004128)

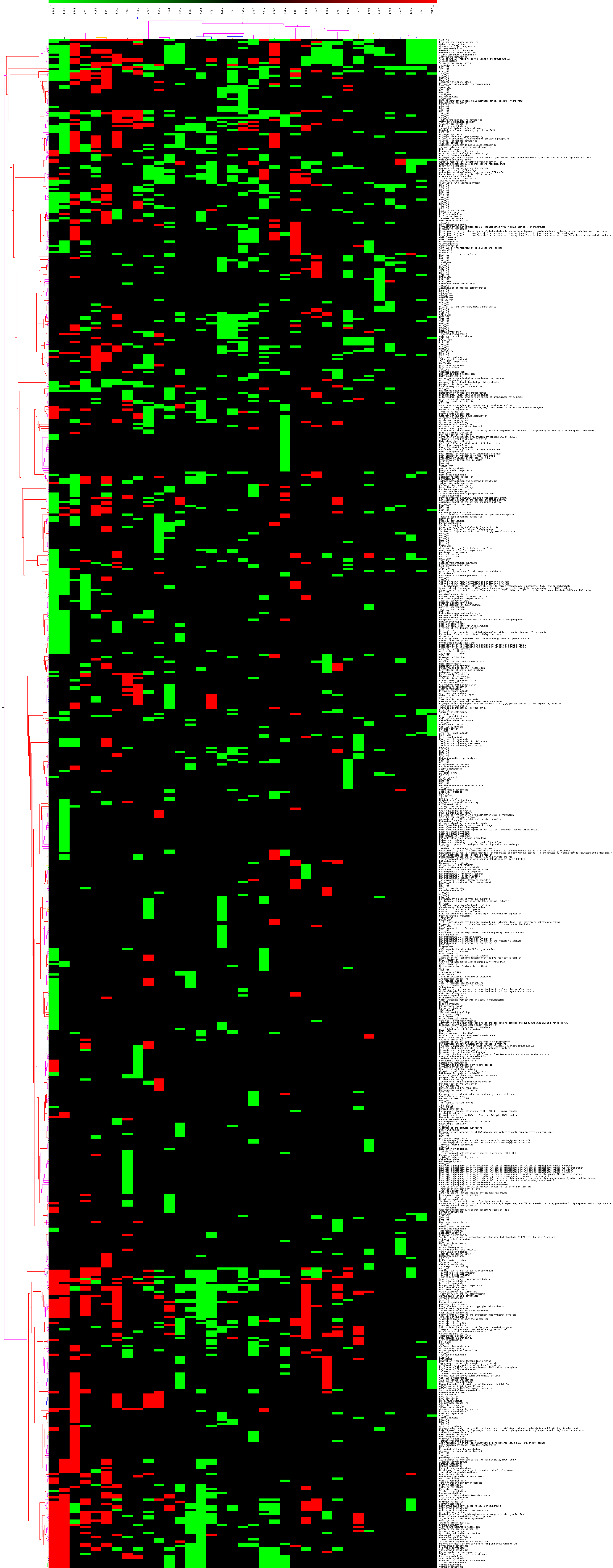

Supplement: Figure S1 — Clustering of signed Binary Enrichment Factors using Euclidean distance using support trees on yeast data. Colored spots indicate significant (p< = 0.05) up- (red) or down- (green) regulation. The colors of the dendrogram indicate the percentages of the tree support (significance), from 50% (pink) to 100% (black). (0.94 MB PNG) [file pone.0004128.s001.png]

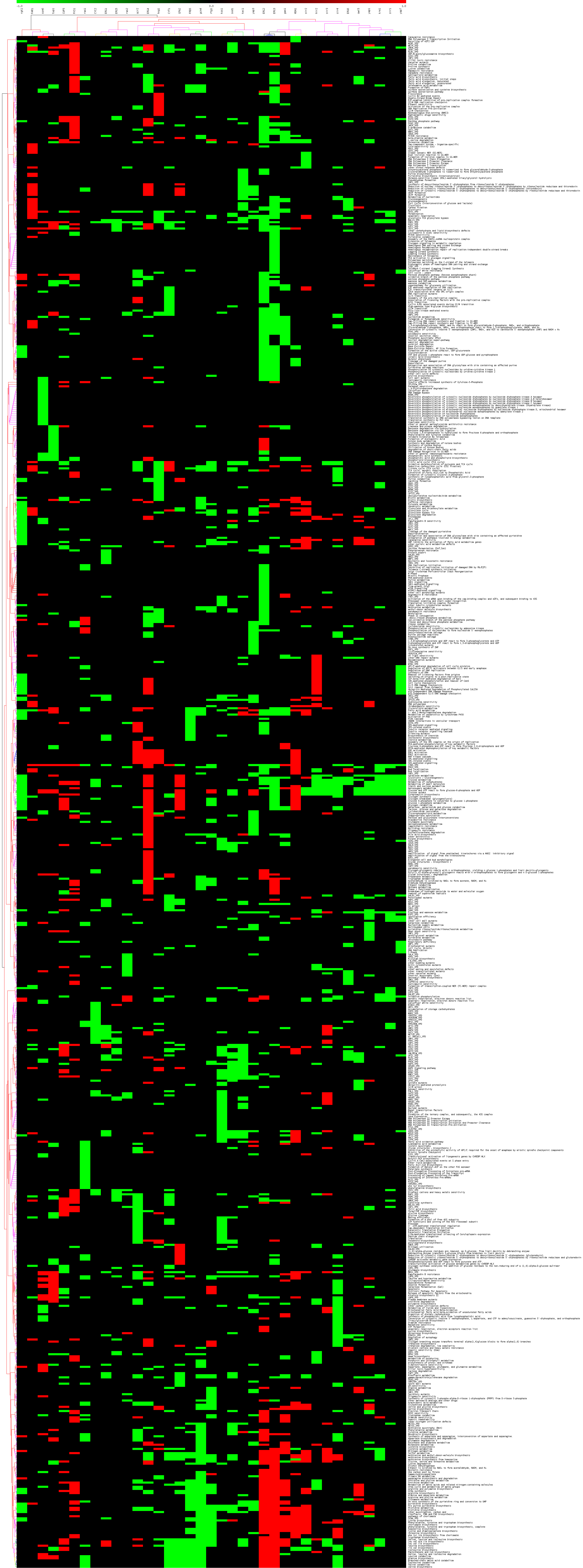

Supplement: Figure S2 — Clustering of signed Binary Enrichment Factors using Pearson's Correlation using support trees on yeast data. Colored spots indicate significant (p< = 0.05) up- (red) or down- (green) regulation. The colors of the dendrogram indicate the percentages of the tree support (significance), from 50% (pink) to 100% (black). (0.92 MB PNG) [file pone.0004128.s002.png]

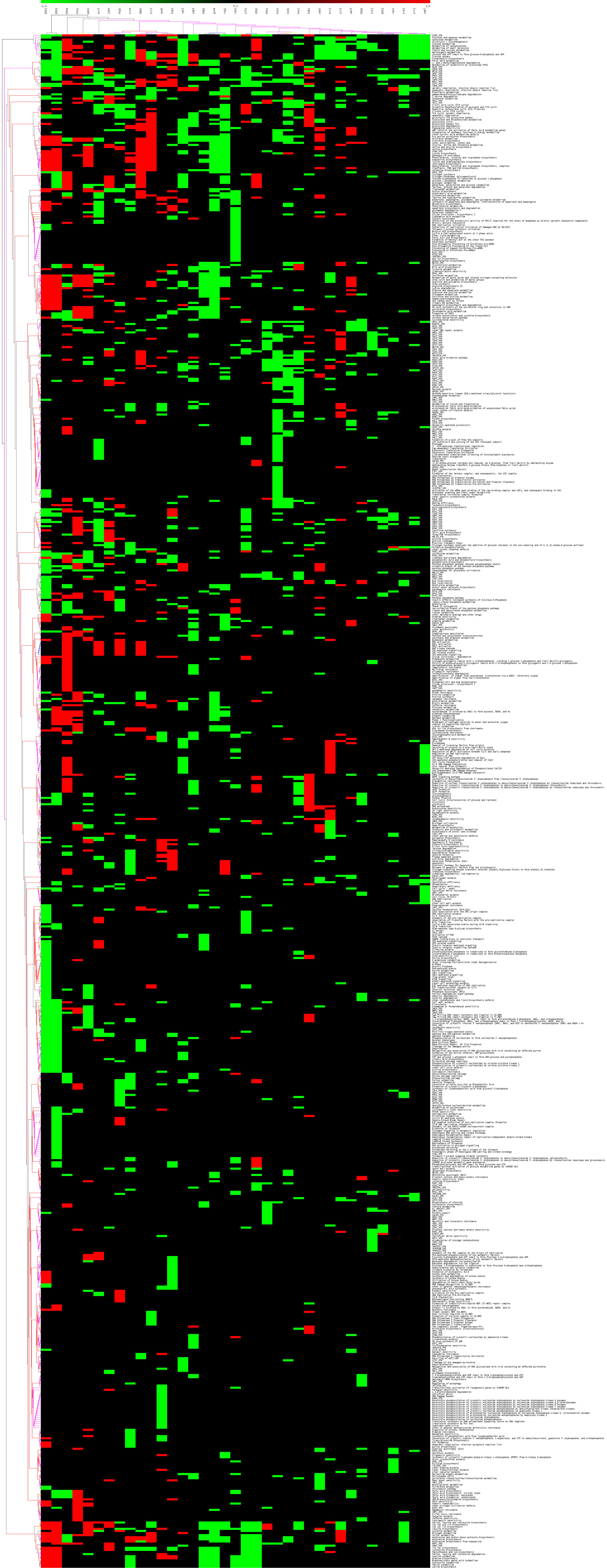

Supplement: Figure S3 — Clustering of signed Binary Enrichment Factors using Manhattan Distance using support trees on yeast data. Colored spots indicate significant (p< = 0.05) up- (red) or down- (green) regulation. The colors of the dendrogram indicate the percentages of the tree support (significance), from 50% (pink) to 100% (black). (0.94 MB PNG) [file pone.0004128.s003.png]

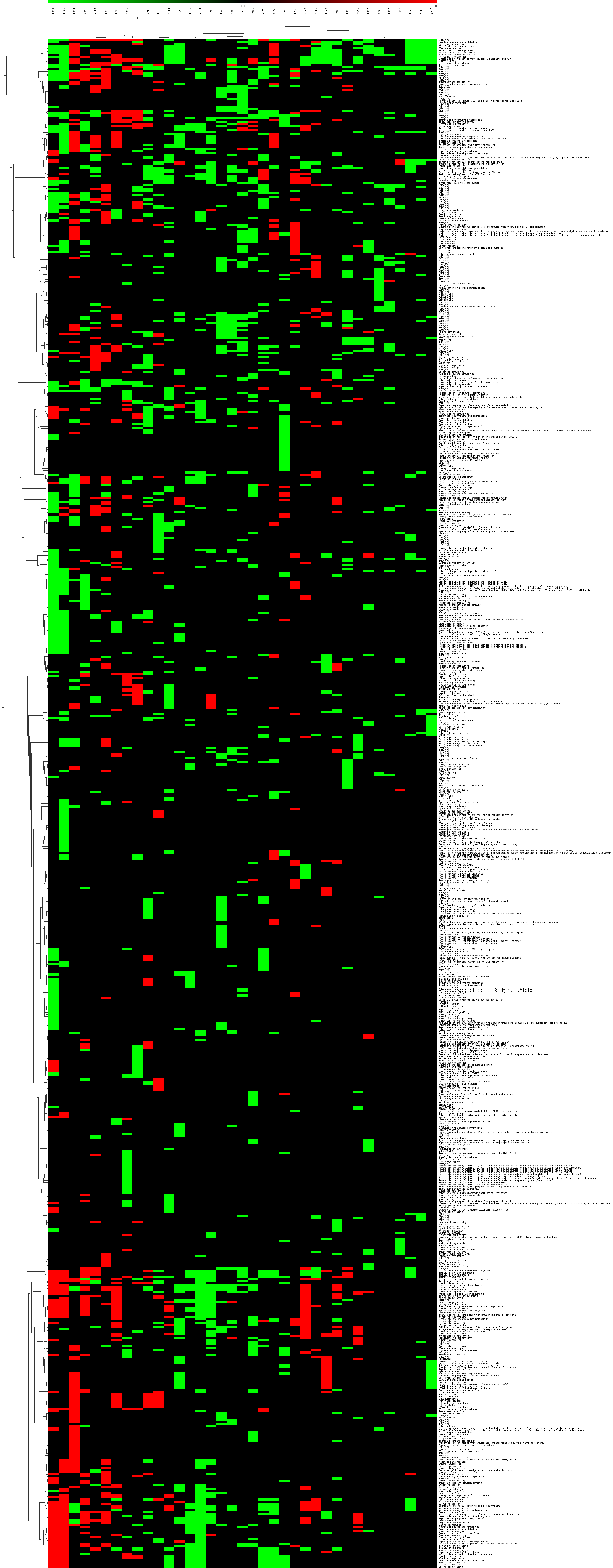

Supplement: Figure S4 — Standard hierarchical clustering of signed Binary Enrichment Factors on yeast data. Colored spots indicate significant (p< = 0.05) up- (red) or down- (green) regulation. (0.92 MB PNG) [file pone.0004128.s004.png]

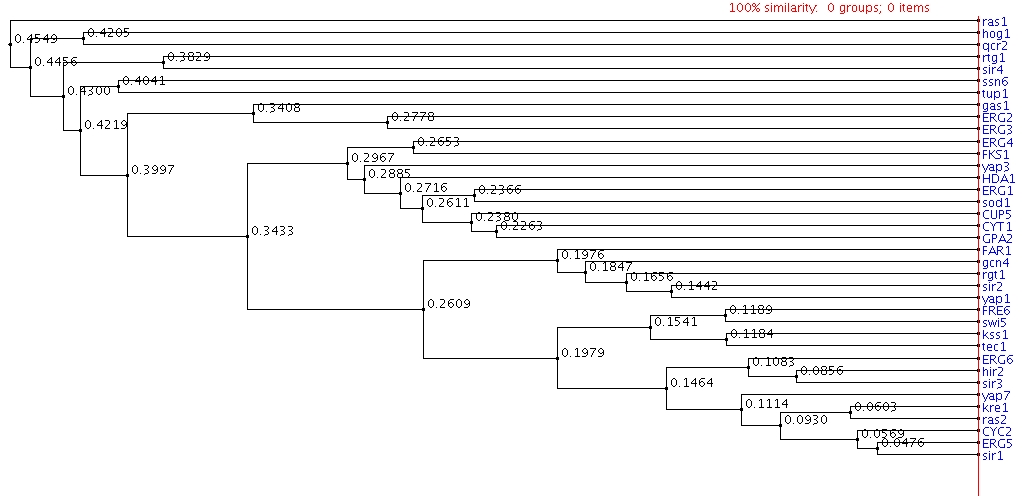

Supplement: Figure S5 — UPGMA clustering of Fisher's Exact Test analysis results on yeast data. (0.16 MB JPG) [file pone.0004128.s005.jpg]

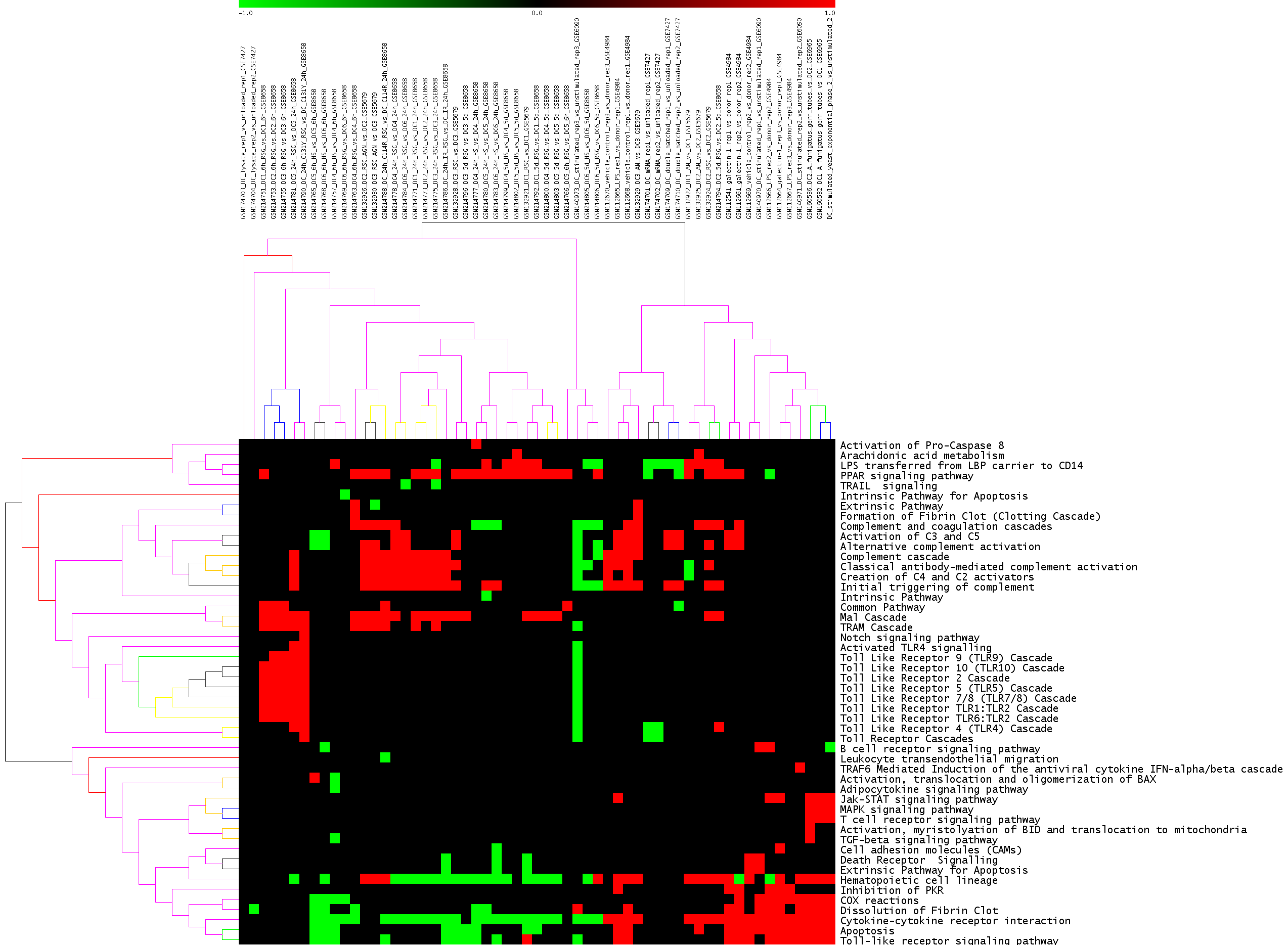

Supplement: Figure S6 — Clustering of signed Binary Enrichment Factors using Pearson's Correlation using support trees on dendritic cell data. Colored spots indicate significant (p< = 0.05) up- (red) or down- (green) regulation. The colors of the dendrogram indicate the percentages of the tree support (significance), from 50% (pink) to 100% (black). (0.22 MB PNG) [file pone.0004128.s006.png]

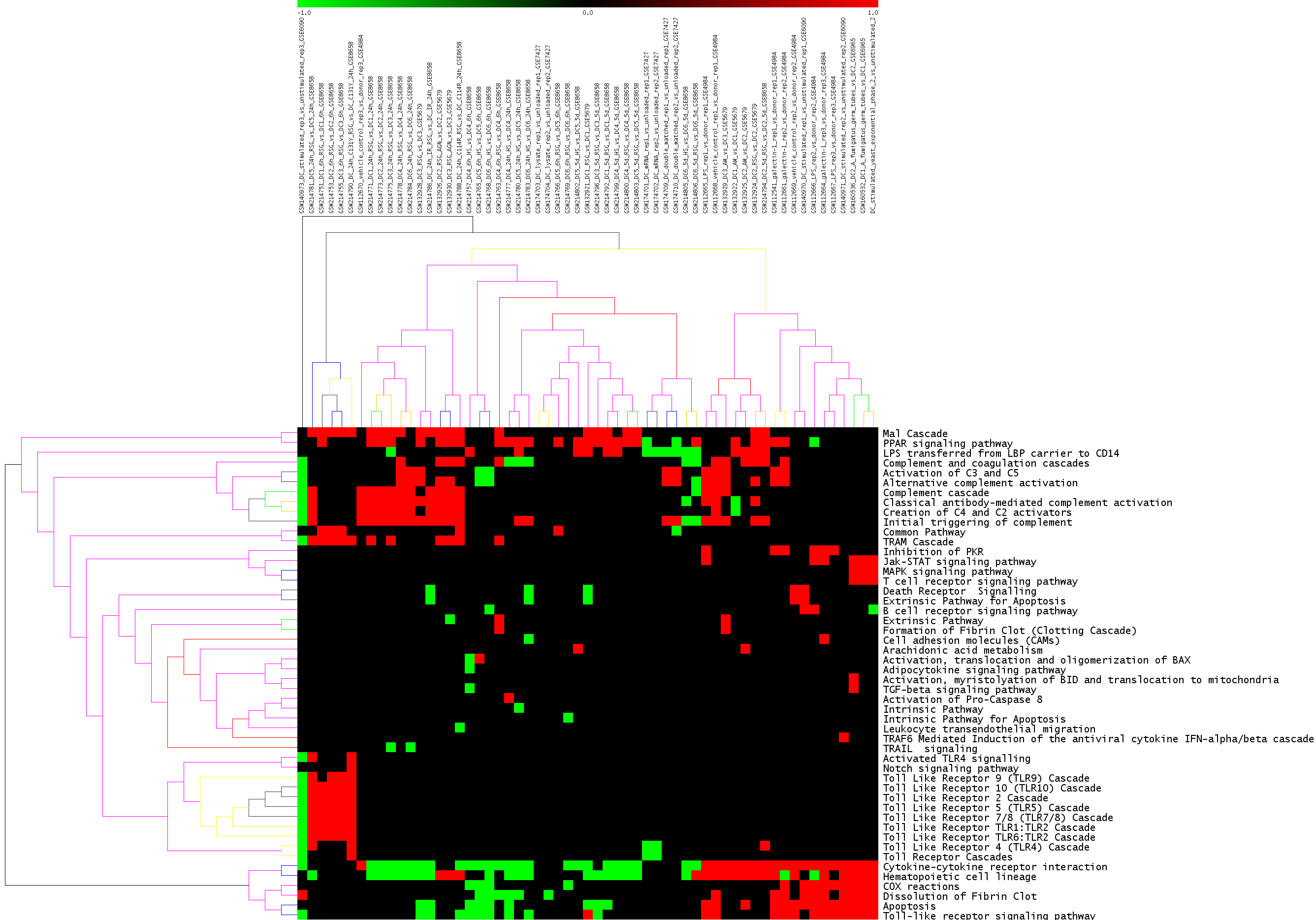

Supplement: Figure S7 — Clustering of signed Binary Enrichment Factors using Manhattan Distance using support trees on dendritic cell data. Colored spots indicate significant (p< = 0.05) up- (red) or down- (green) regulation. The colors of the dendrogram indicate the percentages of the tree support (significance), from 50% (pink) to 100% (black). (0.22 MB PNG) [file pone.0004128.s007.png]

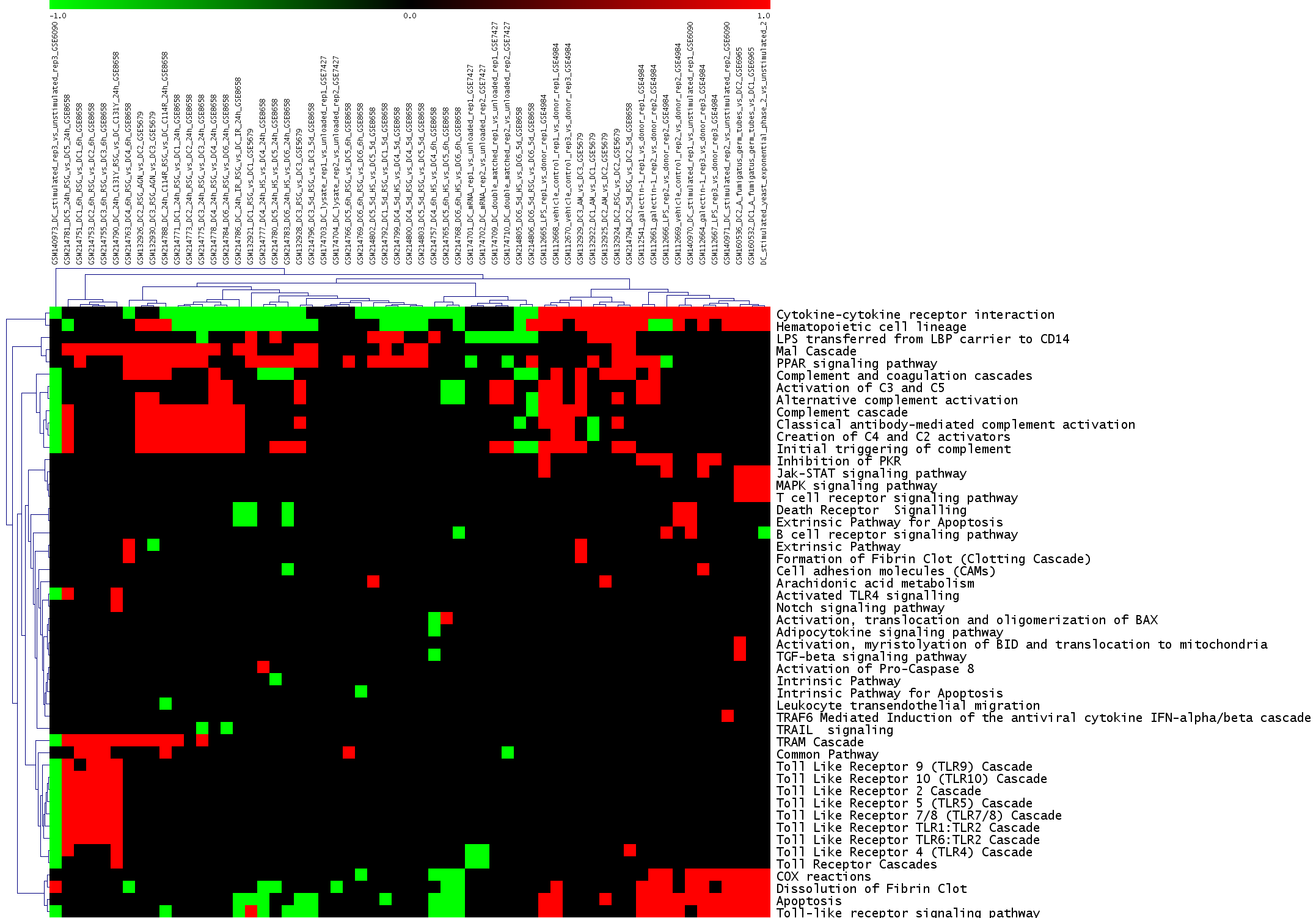

Supplement: Figure S8 — Standard hierarchical clustering of signed Binary Enrichment Factors on dendritic cell data. Colored spots indicate significant (p< = 0.05) up- (red) or down- (green) regulation. (0.20 MB PNG) [file pone.0004128.s008.png]

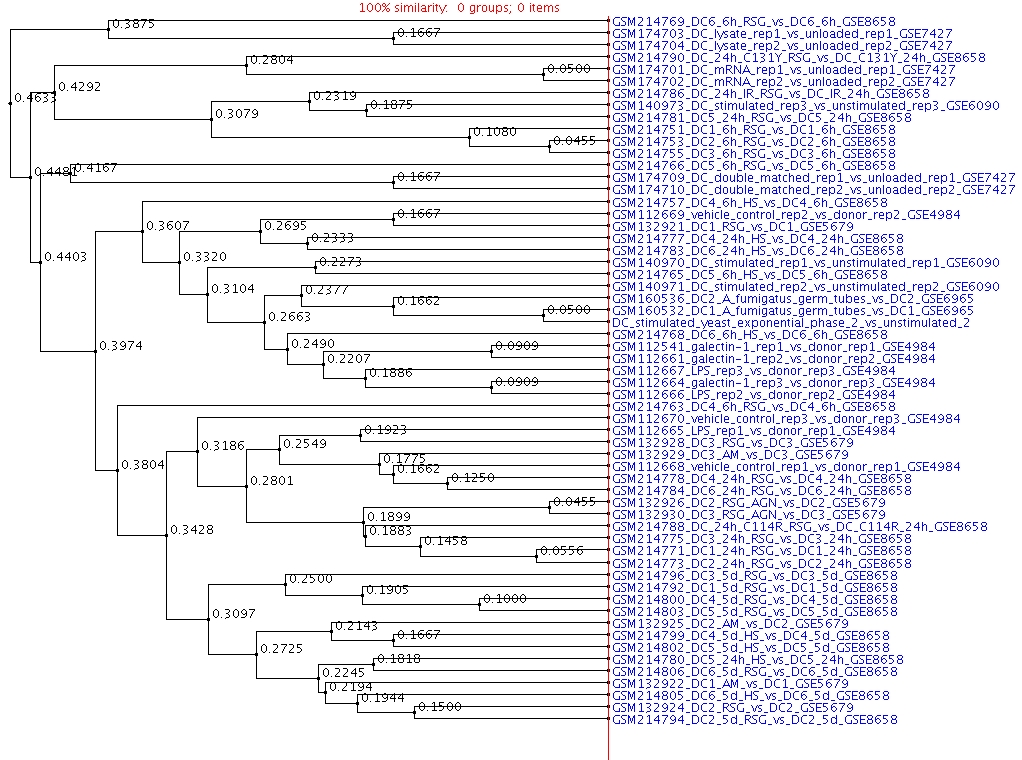

Supplement: Figure S9 — UPGMA clustering of Fisher's Exact Test analysis results on dendritic cell data. (0.63 MB JPG) [file pone.0004128.s009.jpg]
